# The Ventral Anterior Temporal Lobe has a Necessary Role in Exception Word Reading

**DOI:** 10.1093/cercor/bhy131

**Published:** 2018-06-06

**Authors:** Taiji Ueno, Lotte Meteyard, Paul Hoffman, Kou Murayama

**Affiliations:** 1 School of Psychology & Clinical Language Sciences, Centre for Integrative Neuroscience and Neurodynamics, University of Reading, UK; 2 Faculty of Human Sciences, Takachiho University, Tokyo, Japan; 3 Faculty of Environmental Studies, Nagoya University, Nagoya, Japan; 4 Centre for Cognitive Ageing and Cognitive Epidemiology (CCACE), Department of Psychology, University of Edinburgh, Edinburgh, UK; 5 Kochi University of Technology, Kami, Japan; 6 Hector Research Institute of Education Sciences and Psychology, University of Tübingen, Tübingen, Germany

**Keywords:** anterior temporal lobe, computational model, reading, surface dyslexia, transcranial magnetic stimulation

## Abstract

An influential account of reading holds that words with exceptional spelling-to-sound correspondences (e.g., PINT) are read via activation of their lexical-semantic representations, supported by the anterior temporal lobe (ATL). This account has been inconclusive because it is based on neuropsychological evidence, in which lesion-deficit relationships are difficult to localize precisely, and functional neuroimaging data, which is spatially precise but cannot demonstrate whether the ATL activity is necessary for exception word reading. To address these issues, we used a technique with good spatial specificity—repetitive transcranial magnetic stimulation (rTMS)—to demonstrate a necessary role of ATL in exception word reading. Following rTMS to left ventral ATL, healthy Japanese adults made more regularization errors in reading Japanese exception words. We successfully simulated these results in a computational model in which exception word reading was underpinned by semantic activations. The ATL is critically and selectively involved in reading exception words.

## Introduction

Since seminal work by Poljak (Poljak 1926) argued for dual pathways (i.e., 2 mechanisms) for the central auditory system, various cognitive functions have been linked with a dual-pathway neural framework (e.g., vision, motor, attention). Reading is a skill that has to be learnt, so it must piggy-back on existing cognitive and cortical architecture. Indeed, pioneering work(Marshall and Newcombe 1973) in the 1970s explicitly stated the necessity of 2 mechanisms in reading: sublexical processing, which relies on the statistical relationships (or “rules”) between letter(s) and sounds, and whole-word processing, which computes pronunciations from item-specific information (e.g., lexical-semantic knowledge). Reading a word with a regular or typical spelling-to-sound correspondence (e.g., MINT) can rely on the statistical relationships between the sensory information (letters) and the sound/motor information involved in its pronunciation. Contemporary neuropsychological and neuroimaging research agrees that this mechanism is served by the arcuate fasciculus and inferior parietal lobe (e.g., supramarginal gyrus, angular gyrus, and temporoparietal junction) (Jobard et al. 2003; Richlan 2012; Sliwinska et al. 2012; Taylor et al. 2013; Graves et al. 2010, 2014; Hoffman et al. 2015). In contrast, reading a word with an exceptional/atypical spelling-to-sound correspondence (e.g., PINT) cannot rely solely on the spelling-to-sound statistical relationships/rules; this would lead to a regularization error (e.g., reading PINT to rhyme with MINT). Instead, an exceptional item-specific pronunciation must be generated from the whole word. The cognitive and neural architecture that underpins exception word reading remains contentious. We addressed this issue by using a repetitive transcranial magnetic stimulation (rTMS) and computational modeling.

The importance of exception word reading as a window onto human language has been made salient by the neuropsychological and neuroimaging literature. Specifically, the syndrome of semantic variant of primary progressive aphasia (PPA), also known as semantic dementia, is associated with inferolateral temporal cortex atrophy, which is more severe in the anterior relative to the posterior part (Gorno-Tempini et al. 2011). Patients with this condition experience profound loss of verbal and nonverbal semantic knowledge (Patterson et al. 2006, 2007; Woollams et al. 2007; Mion et al. 2010; Shimotake et al. 2015). Crucially, patients with semantic variant PPA not only show impairments for semantic tasks such as object recognition and single-word comprehension, but they also have a difficulty in reading exception/atypical words (Woollams et al. 2007). Moreover, those words are often incorrectly pronounced to be in line with typical spelling-to-sound correspondences (e.g., PINT is rhymed with MINT), also known as “surface errors.” These results suggest that the inferolateral temporal lobe may be crucial for reading exception/atypical words.

The most atrophic area in semantic variant PPA is the ventral part of the anterior temporal lobe (ATL) (Gorno-Tempini et al. 2011), thus some authors have assumed that the ATL plays a necessary role in representation of semantic knowledge and in exception/atypical word reading (Patterson et al. 2006; Woollams et al. 2007; Brambati et al. 2009). This assumption is central to one particular model of reading, the triangle model, which holds that reading words with exceptional spelling-sound mappings is achieved through activation of semantic knowledge (Plaut et al. 1996; Harm and Seidenberg 2004; Woollams et al. 2007; Dilkina et al. 2008). However, due to the progressive, neurodegenerative nature of semantic variant PPA, cortical atrophy is diffuse (Gorno-Tempini et al. 2011) and other temporal lobe regions also show atrophy. Indeed, other researchers have suggested that atrophy in posterior temporal regions may be responsible for the poor exception word reading in this disorder (Coltheart et al. 2010; Richlan 2012; Ripamonti et al. 2014). Thus, semantic variant PPA does not provide a conclusive answer to the question of ATL involvement in exception word reading; instead, it is necessary to use techniques with greater spatial specificity in order to demonstrate that this specific area is crucial for exception/atypical word reading.

Functional neuroimaging studies can potentially address this spatial specificity issue, but results from these studies are also inconclusive. Some studies have associated exception/atypical word reading with ATL activation (Graves et al. 2010; Wilson et al. 2012; Taylor et al. 2013; Hoffman et al. 2015; Provost et al. 2016). However, BOLD responses are always a correlation of the behavior, that is, it can certainly identify the causal effect of stimulus on brain activation, but we cannot derive the effect of a brain activation on behavior. For example, it is difficult to know if the activation is early in cognitive process (i.e., highly likely to be necessary for that process), or caused by some later/downstream process. This is why we need to show a resultant behavior change after the brain activity was causally manipulated, as well as targeting specific cortical regions. Insights on this issue can be gained by using a neurostimulation technique (e.g., rTMS) to temporarily disrupt neural activity in healthy participants (Walsh and Cowey 2000). The effective area of the rTMS pulse is more focal than the diffuse and often extensive damage seen in neurological disease. In addition, an inhibitory effect of rTMS is temporary and is induced in healthy participants, thereby minimizing any plasticity-based recovery that could be seen in patients with long-standing neurological disorders. Related to this, the TMS stimulation temporally precedes an associated behavioral change, and therefore a necessary role for a stimulated brain region can be inferred from the rTMS effect. Here, we would like to make it clear what we mean by “necessary.” We use it in the same manner as is commonly used in cognitive neuroscience (Price et al. 2006). Thus, if a disruption in region A leads to deficits in exception word reading, then it indicates that region A is necessary for the task performance. In other words, the current study does not mean to rule out the role of other, nontested areas in brain for reading exception/atypical word reading.

**Figure 1.**
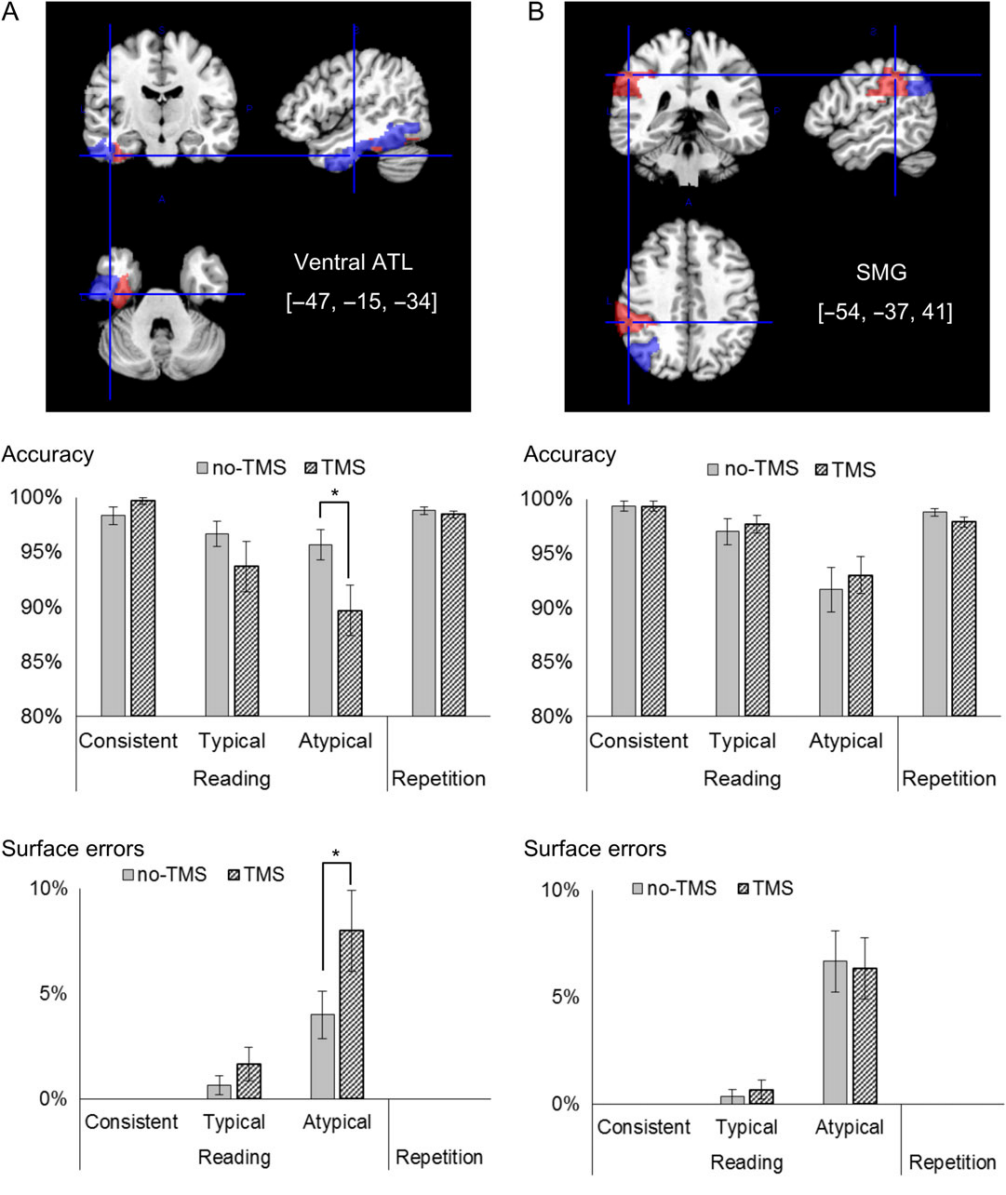
TMS effects on mean accuracy and mean surface errors. (*a*) ATL stimulation site (blue: inferior temporal gyrus; red: fusiform gyrus). (*b*) SMG stimulation site (red: supramarginal gyrus; blue: angular gyrus). Top row: stimulated ROI. Middle row: accuracy. Bottom row: surface reading errors in the main experiment. *P < 0.05. Error bars indicate standard errors See also Figure S1 for individual data plots. See also Figure S2 for the control replication experiment.

The current study directly tested the necessity of the ATL in exception reading by examining the effect of rTMS over this area, compared against several control conditions (cortical site, task, and word sets). We stimulated the ventral anterior inferior temporal gyrus (see top row of Fig. 1), near the anterior fusiform gyrus, based on the recent evidence implicating this precise area in semantic processing (Sharp et al. 2004; Spitsyna et al. 2006; Binney et al. 2010; Mion et al. 2010; Visser et al. 2010; Visser and Lambon Ralph 2011; Shimotake et al. 2015).

Effects in rTMS studies may be caused by global changes in vigilance or task difficulty when TMS is applied. We took 2 approaches to control for these confounds using a within-subjects experimental design. First, oral repetition with the same word set was introduced as a control task. The rTMS effect size on reading was compared with the effect size on this control task. Second, the performance in the ATL–rTMS condition was compared with a no-TMS condition and a control stimulation site (supramarginal gyrus, hereafter SMG: MNI = [−54, −37, 41]). SMG was chosen because this area is well known as a phonological processing area (Fridriksson et al. 2010; Hartwigsen et al. 2010; Richlan 2012; Sliwinska et al. 2014; Pattamadilok et al. 2015) but is not thought to be involved in semantic processing. Thus, we expected that oral repetition would be more sensitive to the stimulation of this site, and reading aloud may be affected more globally rather than a specific effect on exception/atypical words. Potential order and practice effects were checked by replicating the experiment with a separate group of participants without TMS.

Participants were tested for single-word reading and for auditory repetition under rTMS on ATL or SMG on different days. On each day, the control performances in both tasks were evaluated without TMS. The reading materials manipulated consistency and typicality of spelling-to-sound correspondence in Japanese 2-kanji ideograms (consistent words, inconsistent–typical words, or inconsistent–atypical words, see Materials and Methods).

The critical question in the rTMS study was whether ATL–rTMS had a specific effect on exception/atypical words in reading aloud for the group-level analysis (not a subgroup of the participants, c.f., Woollams et al. 2017). To explore the underlying cognitive basis for this effect, we simulated the results of our ATL–rTMS study with a dual-pathway computational model of Japanese reading that incorporates a causal role for semantic processing in word reading. The simulation was motivated by past studies suggesting the ATL supports semantic representations (Harm and Seidenberg 2004; Patterson et al. 2006, 2007; Binney et al. 2010; Mion et al. 2010; Shimotake et al. 2015), and that these are the mechanism by which the correct pronunciation of exception-atypical words is accessed (e.g., triangle model, Plaut et al. 1996; Harm and Seidenberg 2004; Woollams et al. 2007; Dilkina et al. 2008; Welbourne et al. 2011). So, in order to demonstrate the plausibility of this cognitive account for exception/atypical word reading, we tested whether damage to the semantic reading route in our model would produce similar effects to those observed following ATL–rTMS. The simulation strategy will be explained further at the beginning of the simulation results.

## Materials and Methods

### Participants

Overall, 16 native Japanese speakers, living in the UK (for a short-term visit, short-term working-holiday, a study in a university, or a short-term post in a UK branch of Japanese companies), were recruited from London or Reading, UK (12 females and 4 males). One male participant resigned before he completed the full experiment. The mean age was 28.2 (Standard deviation [SD] = 3.91). All were right-handed. The inclusion/exclusion criteria were as follows: 1) participant’s native language was Japanese; 2) normal or corrected to normal vision; 3) normal hearing/no need for hearing aids; 4) they satisfied the commonly accepted inclusion criteria for TMS study, as used worldwide (Rossi et al. 2009); and 5) they passed the internal screening criteria for MRI and TMS studies (e.g., absence of contraindications such as neurological conditions or metal in the head or body) used at the University of Reading. All participants satisfied these criteria. The experiments were reviewed and approved by the local ethics board at the University of Reading (UREC 14/26). All the participants provided full, written informed consent for the MRI study and for the rTMS study at the beginning of every session.

### Reading Stimuli and Task Procedure

The main task was oral reading of 60 single Japanese 2-kanji ideograms (Fushimi et al. 1999). To increase the sensitivity to an ATL–rTMS effect, we tested reading of Japanese kanji words in Japanese speakers. The vast majority of studies of exception/atypical word reading have used English language materials. While there are many exception/atypical words in English, there is still systematicity between orthography and sound. In English, the pronunciations given to vowels in irregular words (e.g., PINT) have correspondences with other words (e.g., PINE). The situation in Japanese is much less systematic. The pronunciation of characters across contexts are often completely unrelated (e.g., the pronunciation of 指, meaning “finger,” may be /sa/, /shi/, or /yubi/ when used in a 2-kanji ideogram, depending on the identity of the adjacent character). In such an opaque language, the contribution of word-specific knowledge is likely to be more crucial, such that an ATL–rTMS effect may be more easily detected.

Further, we used low-frequency words as these are thought to rely more heavily on whole-word knowledge for pronunciation (Plaut et al. 1996; Patterson et al. 2006) and a recent study found that TMS effects in reading are stronger for low-frequency words (Duncan et al. 2010). We used 60 low-frequency 2-kanji materials (Fushimi et al. 1999). This material set has been successfully used to test reading consistency and typicality effects both in neuropsychological and in psychological studies for Japanese-speaking participants (Fushimi et al. 1999). The mean word frequency (log10-transformed occurrences per approximately per a million) was 0.77.

The 60 single Japanese 2-kanji ideograms we used (Fushimi et al. 1999) manipulated consistency and typicality of spelling-to-sound correspondence. Specifically, like vowels in English, kanji characters are an example of a quasiregular domain (Plaut et al. 1996). One-third of kanji characters have only one possible pronunciation (i.e., a fully consistent mapping from orthography to phonology). For example, in the case of 医局 (meaning a medical office), each character has only one possible pronunciation when it appears in each letter position. Thus, this 2-kanji ideogram is categorized as a “consistent” word. In contrast, the remaining two-thirds have several legitimate pronunciations (thus, “inconsistent”). For example, the pronunciation of 指 (meaning finger) may be /sa/, /shi/, or /yubi/ when used in a 2-kanji ideogram, depending on the identity of the adjacent character(s). This is analogous to the different pronunciation of the letters “oo” in the English words “blood” and “hoot,” although the degree of systematicity is even lower in Japanese. Given that many kanji characters have several legitimate pronunciations, many characters have “typical/regular or atypical/exceptional” pronunciations in terms of their type frequency. The level of regularity/typicality of a 2-kanji ideogram has been operationalized by Fushimi et al. (1999). For example, when 食 (meaning eat) appears in the second position of a 2-kanji ideogram, then in most cases the target pronunciation is /syo-ku/ (e.g., 和食 /wa-syo-ku/, meaning Japanese food, 洋食/yo-u-syo-ku/, meaning Western food). This is referred as the typical pronunciation of 食. Both the 2 kanji letters in the inconsistent–typical 2-kanji ideogram condition satisfied this criterion (i.e., most typical). In contrast, there are a few words in which the pronunciation is /ji-ki/ (e.g., 断食/da-N-ji-ki/, meaning fasting). This is referred as an atypical pronunciation of 食. Both the 2 kanji letters in the inconsistent–atypical 2-kanji ideogram condition satisfied this criterion. The mean friends/neighbors ratio (the number of items in a target word’s orthographic neighborhood for which the pronunciation of the component character is the same, divided by all items in the neighborhood) was 1.00 (SD = 0) for the consistent words, 0.71 (SD = 0.07) for inconsistent–typical words, 0.33 (SD = 0.15) for inconsistent–atypical words. Readers are referred to Fushimi et al. (2009) for the whole descriptive statistics for the psycholinguistic variables in this material set. To minimize the burden on participants and to complete all the tasks within the duration of the rTMS effect (approximately 10–15 min), 60 low-frequency items were used (20 items for each of the consistent word condition, inconsistent–typical condition, and inconsistent–atypical condition). The presentation of each word type was fully randomized across participants by using the random seeds that were generated by the experimental program.

Each trial began with a beep sound and with a presentation of a warning cross for 500 ms, followed by a presentation of a 2-kanji ideogram at the center of the screen. The font size was 200, and the font style was Gothic. The stimulus remained on screen until the next trial began (5 s later). During this 5-s time window, participants were required to read aloud as correctly and quickly as possible. A PC running HSP3.0 (Hot Soup Processor: http://hsp.tv/) was used for the presentation of stimuli. Reaction time was measured from the stimulus onset until the beginning of an oral response by using a voice key. Only RTs for correct trials were submitted to the analysis.

### Repetition Stimuli and Task Procedure

Oral repetition was introduced as a control task. The same lexical items as reading were used to control the possible confounds of item-specific effects. The auditory stimuli for these lexical items were taken from NTT database (Amano and Kondo 2000). In each auditory file, the silent time window before the onset of the word was removed using Audacity software (http://audacity.sourceforge.net).

Each trial began with a beep sound and a presentation of a warning cross at the center of the screen for 500 ms, followed by an auditory presentation of a 2-kanji ideogram via earphones. The next trial began 5 s later. During this 5-s window, participants were required to repeat aloud as correctly and quickly as possible. The order of reading and repetition tasks were counterbalanced across participants.

### MRI and rTMS

In the rTMS condition, immediately before the reading/repetition tasks, the participants received 10-min of 1 Hz (90% resting motor threshold) rTMS. TMS was delivered with a Magstim Standard Rapid magnetic stimulator fit with a 70-mm figure-8 stimulating coil (Magstim Co., Whitland, UK). The regions of interest (ROI) were the left ventral ATL (*x* = −47, *y* = −15, *z* = −34), and the left supramarginal gyrus (SMG: *x* = −54, *y* = −37, *z* = 41). The co-ordinate of the ATL was determined, based on the recent evidence implicating this precise area in semantic processing (Sharp et al. 2004; Spitsyna et al. 2006; Binney et al. 2010; Mion et al. 2010; Visser et al. 2010; Visser and Lambon Ralph 2011; Shimotake et al. 2015). Specifically, the *z*-axis co-ordinate was set to the averaged value of the peak-points reported in these studies. The *x*-axis co-ordinate was set to the most lateral one of these studies as TMS pulse may be difficult to reach more medial areas. The *y*-axis co-ordinate was set to the most posterior one (yet within the ATL) in order to minimize the uncomfortable muscle-twitches in the face due to TMS pulse. The co-ordinate of the SMG was determined, based on the recent TMS study that probed the phonological function of this site (Pattamadilok et al. 2015). Accurate targeting of each ROI was achieved by obtaining a high resolution anatomical MRI of each subject with a 3-T scanner (A Siemens Magnetom Trio, 256 saggital slices, 1.0 mm × 1.0 mm × 1.0 mm) and using an infrared-based frameless stereotaxy system (Brainsight TMS Navigation, Rogue Resolutions Ltd, Cardiff, UK).

### Testing Schedules

Each participant completed 2 sessions on different days (separated by least 3 days) with stimulation of either ATL or SMG on each day. On each day, both the reading task and the repetition task were conducted twice: one immediately after rTMS and the other without TMS (i.e., control performance). We define the effect size of rTMS at each site as the RT or accuracy difference between the rTMS condition and the no-TMS condition conducted on the same day. The order of rTMS and no-TMS conditions was counterbalanced to control for practice effects. When the rTMS condition preceded the no-rTMS condition, the latter was started at least 30 min after the delivery of the rTMS pulses.

### Control Experiment

In addition to randomly determining the orders of each condition, we examined practice effects by conducting a follow-up control experiment with all the same orders of the conditions/materials (i.e., yoked-control) as the main experiment but without delivering an rTMS pulse in the “rTMS condition.” This way, we confirmed that the performances in the “rTMS condition” did not differ from the “no-rTMS condition” in this control experiment (see Supplementary Materials for the results). The same number of participants as the main experiment were collected for this control experiment, from Nagoya University (12 females and 3 males, mean age = 25.00, SD = 3.11).

### Statistics and General Methods in Human Experiment

#### Sample Size and Randomization

The sample size was chosen as follows: First, we used Google Scholar, and retrieved 111 articles (retrieved 1 July 2013) which cited the first rTMS study on left ATL function (Pobric et al. 2007). Among these 111 articles, 7 articles investigated the left ATL–rTMS effect on conceptual or language tasks with a within-subjects design. A random-effects meta-analysis of these articles (13 dependent measures) resulted in an integrated effect size of Cohen’s *d* = 1.382 (0.779 1.985). A power analysis on this integrated effect size revealed that a sample size of 11 would give power of more than 0.999. Next, in order to satisfy the complete counterbalancing in the order of the conditions, the sample size should be a multiple of 8. Taken together, we therefore determined 16 as the target sample size. Each participant was randomly allocated (i.e., in the order of their participation to the study) to 1 of the 8 possible order patterns. Except for the experimenter, who knew in which order each participant took each condition, all the other investigators were fully blinded to this allocation.

#### Data Availability

All the human data and the simulation code are available via e-mail request.

### Computer Simulation

#### Architecture and Connectivity

The neural network model incorporated 4 processing systems (visual input system, phonetic input system, semantic input/output system, and phonetic output system). With these systems, the model was trained 1) for reading (visual → phonology), 2) for kanji comprehension (visual → semantics), 3) for speaking (semantics → phonology), and 4) for repetition (phonology → phonology). The architecture and connectivity of these 4 systems was based broadly on the neuroanatomy of the 4 areas in the brain (i.e., a visual processing area, an auditory processing area, ATL, and inferior frontal cortex). Since it is unlikely that these 4 areas in the brain are connected directly, the intermediate layers (so-called hidden layers) were included between each processing systems.

#### Representations

The model was trained on a large set of Japanese 2-kanji ideograms and its performance was then assessed on a set of test items. To compare the model’s performance to the human data, the test items were the same 120 2-kanji words from Fushimi et al. (1999). For the sake of brevity, the training items were restricted to all the Japanese 2-kanji ideograms which include at least one of the kanji character used in the 120 words testing set. As a result, 9818 items were extracted from the NTT database (Amano and Kondo 2000) and used for training. The input pattern for reading was bitmap images of 2 kanji letters (see later). The target pronunciation (i.e., target phoneme/mora sequence) for reading was coded in terms of the set of distinctive phonetic features for each phoneme/mora (e.g., sonorant, nasal). The full detail of these patterns was reported elsewhere (Ueno et al. 2011; Ikeda et al. 2016). The same distinctive phonetic pattern was used for the input of the auditory repetition task. Semantic representations (i.e., target for word comprehension, or input for speaking) were abstract vector patterns of 100 bits, which captured the core features of human conceptual knowledge (e.g., semantic category, arbitrary mapping to phonology). Given the partial systematicity between orthography and semantics in Japanese kanji, such a relationship was implemented as follows. First, a “prototype” semantic pattern (50 bits) for each kanji character was created by randomly setting 30/50 units to a value of 1. Then, each semantic exemplar (50 bits) was generated from each prototype by randomly setting 10/30 “on” units to a value of 0. Next, the semantic representation (100 bits) for a 2-kanji ideogram was created by concatenating the two 50-bit exemplars for 2-kanji characters. This way, the resultant semantic pattern was partially correlated to its orthographic pattern but was more arbitrary to its phonetic pattern.

#### Training

All the adjacent layers were fully connected bidirectionally. The activity of each unit ($a_{i}$, below) was a sigmoid function of the summed weighted input ($s_{i}$, below) from other units in the following equations.

$$s_{i}=\underset{j}{\sum }w_{j.i}\times a_{j}$$

$${\;a}_{i}=\frac{1}{1+e^{\left(-s_{i\;}\times gain\right)}}$$

where, $s_{i}$ is the net input of unit *i* from all of the projections *j* to the unit *i*, and $a_{i}$ is the activation of the unit *i*, which is a logistic function of the net input, ranging from 0 to 1. The input gain parameter was set to 1. The network was operated in a continuous manner across 20 time steps, during which the time-integrated input value of each unit gradually changes as follows:

$$time\;integrated\;s_{i.t}=s_{i.t-1}+0.1*\left(s_{i.t}-s_{i.t-1}\right)$$

where, the updating weight value (0.1) was a priori determined for a practical reason. If one decreases this value, then a continuous network can be simulated at a finer grain (i.e., higher time-resolution) but it takes more time to train. Given the network size was huge (i.e., number of units and the number of training examples), we refrained from selecting a too small value (e.g., 0.01–0.05).

Lens (http://tedlab.mit.edu/~dr/Lens/) was used for training. Momentum was not used. Cross-entropy was used to estimate the error associated with each unit, and the connection strength was adjusted through the back-propagation algorithm after every trial. The weight update was skipped when the difference between the target value and the output value was below 0.1. The root-squared word frequency count per million was used to scale the error derivatives associated with each word, to simulate the effect of greater training for higher-frequency words. There were 40 epochs of training, each of which contained a presentation of each of the training patterns 20 times in random order.

The training regime for the model reflected the developmental sequence experienced by children in early life. Specifically, the first 20 epochs reflected a preliteracy period, where 95% (19 times presentations within an epoch) of the training was for speaking whereas 5% (single presentation within an epoch) was for repetition. Learning rate and weight decay started at 0.5 and 0.0000005, respectively, and each was reduced by 0.05 and 0.00000005, respectively, per 10 epochs. After this preliteracy training, the weights between the phonetic output layer and the semantic/phonetic input layer were frozen (i.e., error derivatives were set to zero). The next 20 epochs reflected a literacy period, where one epoch consisted of 6 times presentation of each item for reading (30%), of 12 times presentation for word comprehension (60%), of a single presentation for speaking (5%) and of a single presentation for repetition (5%). Learning rate and weight decay started at 0.5 and 0.0000005, respectively, and each was reduced by 0.05 and 0.00000005, respectively, per 2 epochs. Moreover, Japanese children receive heavy drilling in kanji literacy when at school (i.e., they are expected to repeatedly practice writing a kanji and its phonemic/moraic form in a note if they fail this item in a test). To reflect this drilling, a reading test was conducted for the 120 words from Fushimi et al. (1999) after every 2 epochs, and any incorrect items received further training for reading. During this training, the error derivatives associated with each word error was not scaled by its word-frequency. This drilling procedure was reiterated 20 times after every 2 epochs.

#### Testing and Lesioning

During testing, a nearest-neighbor criterion was taken to assess the model’s output. Thus, the word with the closest (in terms of Euclidian distance) phonemic/moraic vector pattern was selected as the model’s output. As a result, reading accuracy in the model was compatible with human data (black marker of Fig. 3*c*).

When simulating the rTMS effect on ATL, approximately 5% of the units in the semantic system layer were randomly selected and were deactivated. Reading accuracy was evaluated after this unit inhibition. This procedure was reiterated 50 times and the resulting accuracies were averaged. The number of units to inhibit was determined so that the resultant reading accuracy for exception/atypical words was matched with human data. The error patterns were coded in the same criterion as the human experiments (see main text). The simulation code is also available via e-mail request.

## Results

### Reading Accuracy

Reading accuracy results, shown in Figure 1, indicated that TMS to the ATL had a selective effect on the reading of atypical words. On reading accuracy (Fig. 1, second row), a 3-way ANOVA (rTMS effect: with-TMS or no-TMS by site effect: ATL or SMG by word-type effect: consistent, typical, or atypical) revealed a nonsignificant main effect of site (*F*[1, 14] = 0.298, *P* = 0.593, n.s., *η*^2^*p* = 0.020), a nonsignificant main effect of TMS (*F*[1, 14] = 1.987, *P* = 0.180, n.s., *η*^2^*p* = 0.124), and a significant main effect of word-type (*F*[2, 28] = 15.838, *P* < 0.001, *η*^2^*p* = 0.530). Importantly, there was a significant 3-way interaction (*F*[2, 28] = 5.077, *P* = 0.013, *η*^2^*p* = 0.266). When stimulating SMG, a 2-way interaction was nonsignificant (rTMS effect by word-type effect: *F*[2, 28] = 0.615, *P* = 0.547, *n.s.*, *η*^2^*p* = 0.042). In contrast, when stimulating ATL, a 2-way interaction was significant (rTMS effect by word-type effect: *F*[2, 28] = 4.534, *P* = 0.019, *η*^2^*p* = 0.244). Although not predicted, rTMS significantly improved accuracy for consistent words (*F*[1, 14] = 5.090, *P* = 0.040, *η*^2^*p* = 0.266). A close inspection of the error patterns in the consistent-word no-TMS condition revealed that the most frequent error pattern was a “semantic error.” Specifically, the participants pronounced as if they read another but semantically similar word from the target (e.g., 理学, meaning science, was pronounced as 美学, meaning aesthetics. Note that the second character 学 is shared by these 2 words, and this character means -logy in Japanese). Interestingly, such “semantic errors” in the consistent condition without TMS (*M* = 1.66%, SE = 0.79%) marginally significantly declined by TMS (*M* = 0%, SE = 0%), *P* = 0.055. Since the overall frequencies of the incorrect responses were very low, only a marginal effect is not surprising, and explainable. The other type of errors was stuttering, and it was observed just once for one participant. So, one possible reason would be a TMS-induced inhibition of semantic errors in reading. It is possible that by inhibiting the role of ATL, the participants focused the spelling-to-sound statistical relationship, which of course led to the accurate response in Consistent condition. One may argue that the error pattern described here could be not just semantic but phonological, as these words share the same kanji character. If so, the same error patterns should be reduced by TMS in word repetition (i.e., a typically dorsal processing route that relies more on phonology). However, in the repetition task, the proportion of the “semantic”/“phonological” errors in the consistent word condition) was not reduced by TMS (before TMS: *M* = 2.33%, SE = 0.66%; after TMS: *M* = 3.33%, SE = 0.79%; *P* = 0.44). Thus, our tentative account would be that the nature of disruption by ATL–TMS is semantic, but further exploration is undoubtfully necessary in the future.

Next, there was no ATL-rTMS effect for inconsistent–typical words (*F*[1, 14] = 1.817, *P* = 0.199, n.s., *η*^2^*p* = 0.114). Importantly, ATL-rTMS significantly impaired reading accuracy for atypical/exception words (*F*[1, 14] = 7.485, *P* = 0.016, *η*^2^*p* = 0.348) as we predicted. This rTMS effect on exception/atypical reading was ATL-specific: there was a significant 2-way interaction (rTMS effect by site effect) on reading exception/atypical words (*F*[1, 14] = 8.406, *P* = 0.011, *η*^2^*p* = 0.375), and the rTMS effect was nonsignificant when SMG was stimulated (*F*[1, 14] = 1.365, *P* = 0.262, n.s., *η*^2^*p* = 0.088).

### Surface Errors in Reading

The impaired reading accuracy in exception/atypical word reading reflected an increase in surface errors (Fig. 1, bottom row). A surface error refers to an output of another, legitimate pronunciation of that character (e.g., PINT is rhymed with MINT) or a generation of the first syllable of a more regular pronunciation followed by stuttering, and then a self-correction. First, there were nonsignificant main effect of site (*F*[1, 14] = 0.011, *P* = 0.910, n.s., *η*^2^*p* = 0.0008), a significant main effect of TMS (*F*[1, 14] = 6.176, *P* = 0.026, *η*^2^*p* = 0.306), and a significant main effect of word-type (*F*[2, 28] = 21.563, *P* < 0.001, *η*^2^*p* = 0.606). Importantly, there was a significant 3-way interaction (rTMS effect by site effect by word-type effect) was significant (*F*[2, 28] = 3.897, *P* = 0.032, *η*^2^*p* = 0.217). When stimulating SMG, a 2-way interaction was nonsignificant (rTMS effect by word-type effect: *F*[2, 28] = 0.318, *P* = 0.730, n.s., *η*^2^*p* = 0.022). Importantly, when stimulating ATL, a 2-way interaction (rTMS effect by word-type effect) was significant (*F*[2, 28] = 3.615, *P* = 0.040, *η*^2^*p* = 0.205). A subsequent analysis revealed there was not a significant effect of rTMS in reading consistent words (*F*[1, 14] = 0), and only a marginally significant effect in typical–consistent word reading (*F*[1, 14] = 3.500, *P* = 0.082, *η*^2^*p* = 0.200). Importantly, as predicted, ATL–rTMS significantly increased the frequency of surface errors in reading exception/atypical words (*F*[1, 14] = 4.732, *P* = 0.047, *η*^2^*p* = 0.252). The generated responses were more regular pronunciations of that character than the target pronunciation. To test whether this rTMS effect on exception/atypical word reading was ATL specific, we conducted a 2-way ANOVA (rTMS effect: with-TMS or no-TMS by site effect: ATL or SMG) on the frequency of surface errors in reading exception/atypical words. As a result, the 2-way interaction was marginally significant (*F*[1, 14] = 4.414, *P* = 0.054, *η*^2^*p* = 0.239). There could be one possible reason for this effect to remain only marginally significant: Specifically, the number of errors in each condition was very low, as such it was not very sensitive to test an interaction effect of 2 variables on the frequency of a specific type of errors among such infrequent errors. Though this is a plausible explanation, since the interaction was nevertheless marginal, we would refrain from a strong argument: For the readers’ information the simple effect of the rTMS effect on surface errors was nonsignificant when SMG was stimulated (right column of Fig. 1: *F*[1, 14] = 0.189, *P* = 0.670, n.s., *η*^2^*p* = 0.013).

### Reading RT

**Figure 2.**
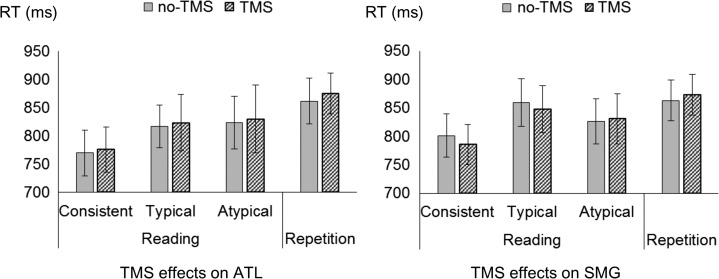
Mean reaction time as a function of the TMS effects on ATL (left column) and on SMG (right column) in the main experiment. Error bars indicate standard errors *P < 0.05. See also Figure S1 for individual data plots.

On reading RT (Fig. 2), a significant rTMS effect was not observed in any condition. First, a 3-way interaction was not significant (rTMS effect by site effect by word type effect: *F*[2, 28] = 0.556, *P* = 0.579, n.s., *η*^2^*p *= 0.038). Also, there was neither a significant 2-way interaction (rTMS effect by site effect) (*F*[1, 14] = 0.154, *P* = 0.699, n.s., *η*^2^*p *= 0.010) nor a 2-way interaction (rTMS effect by word-type effect) (*F*[2, 28] = 0.260, *P* = 0.772, n.s., *η*^2^*p* = 0.018). The main effect of rTMS was not significant either (*F*[1, 14] <0.001, *P* = 0.987, n.s., *η*^2^*p* <0.00001). The main effect of site was nonsignificant (*F*[1, 14] = 0.474, *P* = 0.502, n.s., *η*^2^*p* = 0.032), and that of word-type was significant (*F*[2, 28] = 20.275, *P* < 0.001, *η*^2^*p* = 0.591). Hence, in our study, accuracy seemed to be more sensitive to the TMS effect rather than RT, as in some previous rTMS studies (Whitney et al. 2011).

### Repetition Accuracy

Performance in the repetition task is shown in the right edge of each panel in Figures 1 and 2. On repetition accuracy (Fig. 1, second row), 2-way ANOVA (rTMS effect by site effect) did not reveal a significant interaction (*F*[1, 14] = 1.206, *P* = 0.290, n.s., *η*^2^*p* = 0.079). Neither the main effect of rTMS (*F*[1, 14] = 3.057, *P* = 0.102, n.s., *η*^2^*p* = 0.179) nor the main effect of site was significant (*F*[1, 14] = 1.000, *P* = 0.334, n.s., *η*^2^*p* = 0.066). For readers’ information, the simple effect of rTMS on SMG was marginally significant on repetition accuracy (*F*[1, 14] = 3.796, *P* = 0.071, *η*^2^*p* = 0.213), and repetition accuracy was lower under SMG–rTMS than without TMS. In contrast, the ATL–rTMS effect was not significant (*F*[1, 14] = 0.677, *P* = 0.424, n.s., *η*^2^*p* = 0.046).

### Repetition RT

Like reading RT, repetition RT (Fig. 2) was not sensitive to the TMS effect in our study. There was not a significant 2-way interaction (rTMS effect by site effect) (*F*[1, 14] = 0.024, *P* = 0.883, n.s., *η*^2^*p* = 0.001). Neither the main effect of TMS (*F*[1, 14] = 0.476, *P* = 0.501, n.s., *η*^2^*p* = 0.032) nor the main effect of sites was significant (*F*[1, 14] = 0.003, *P* = 0.956, n.s., *η*^2^*p* = 0.0002).

### Control Experiment

We checked potential order and practice effects (e.g., interaction between the order of items and the order of conditions) by replicating the experiment with a separate group of participants without TMS (with all the same orders of the conditions/materials, that is, yoked-control procedure). This replication study did not find a significant difference between accuracies in the “rTMS” condition (no TMS) and in the no-TMS condition (see Supplementary section). Thus, the accuracy decline in the main experiment was TMS-specific, not being confounded with other potential order and practice effects

### Computational Modeling

**Figure 3.**
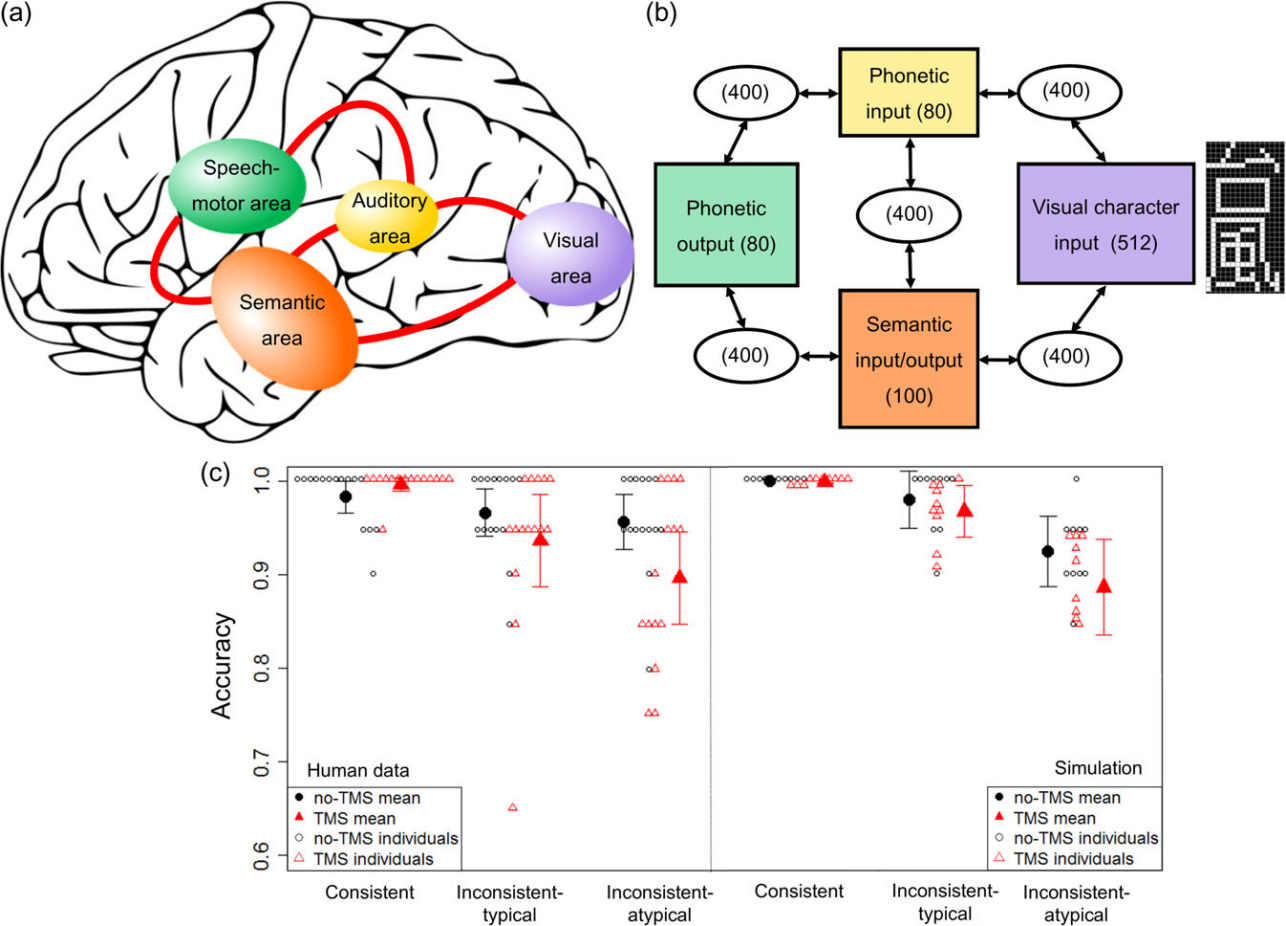
Computational modeling. (*a*) Dorsal–ventral dual-pathway model for reading that incorporates the role of semantics in ATL (Hoffman et al., 2015): implemented neural network model for dorsal–ventral dual-pathway model (with number of units in the parentheses). (*c*) TMS effects on reading accuracies in the normal participants (taken from Fig. 1) and simulated performance under lesioning of semantic activations in the model. Error bars indicate standard errors In the implemented model (panel *b*), each primary systems (i.e., colored layers) were connected via hidden layers.

The rTMS evidence above supports the necessary role of the ATL in exception/atypical word reading. Past studies suggest the role of ATL in semantic representations, which is in keeping with the hypothesis that correct reading of exception words is achieved through access to semantic knowledge (Patterson et al. 2006, 2007; Woollams et al. 2007; Mion et al. 2010; Shimotake et al. 2015). To test whether this view could account for our findings, we simulated the results of our ATL–rTMS study with a dorsal–ventral dual-pathway computational model of Japanese reading that incorporates a role for semantics (Fig. 3*a*,*b*, see Materials and Methods).

The performance of the trained model, with and without disruption to semantic units, is shown on the right-hand side of Figure 3*c*. The same number of simulations as the human participants (see above) were run with different initial weight matrices, and the results were averaged. After training, reading accuracy of the model (black markers) was comparable to human participants (left-hand side of Fig. 3*c*). When the unit activities in the semantic system were inhibited (an approximation of ATL–rTMS), then reading accuracy for exception/atypical words were significantly impaired in the model (red markers in Fig. 3*c*), (effect of semantic lesioning in exception words: *F*[1, 14] = 13.609, *P* = 0.002, *η*^2^*p* = 0.492). In contrast, reading accuracy for typical words did not decline significantly (effect of semantic lesioning in typical words: *F*[1, 14] = 3.660, *P* = 0.076, *η*^2^*p* = 0.207). The 2-way interaction was also significant (typicality by semantic lesioning: *F*[1, 14] = 11.235, *P* = 0.004, *η*^2^*p* = 0.445). Moreover, this impaired accuracy for exception/atypical words by lesioning reflected a significantly increased rate of surface errors. Specifically, there was a significant 2-way interaction (effect of semantic lesioning by effect of word-type [typical or atypical]: *F*[2, 28] = 25.629, *P* = 0.0002, *η*^2^*p* = 0.646). The surface error increased for reading exception/atypical words (from 0.02% → 2.32%) (effect of semantic lesioning: *F*[1, 14] = 25.750, *P* = 0.0002, *η*^2^*p* = 0.647) but only marginally significantly increased for reading typical words (from 0.00% → 0.5%) (effect of semantic lesioning: *F*[1, 14] = 3.500, *P* = 0.082, *η*^2^*p* = 0.200).

## Discussion

Past reading theories have assumed the role of lexical-semantic representations in reading exception/atypical words, and neuropsychological and neuroimaging literature points to a role for the left ATL for this cognitive processing. However, it has been unclear whether the ATL has a necessary role in reading exception/atypical words. One reason for this uncertainty lies in the difficulty in interpreting evidence from progressive neurological cases, due to the diffuse brain damage and the possibility of plasticity-based recovery. Neuroimaging evidence provides an important additional insight but it was necessary to test whether disruption to the function of the area leads to disruption in behavior. We used rTMS to temporarily inhibit a focal region of the ATL, which various neuroimaging and neuropsychological studies suggest has a crucial role in lexical-semantic processing, and found that exception/atypical word reading was less accurate. In addition, participants made a higher number of surface errors. Since these errors are the same as those produced during exception word reading in the semantic variant of PPA, this bolsters the interpretation the targeted area in ATL by our TMS study has a necessary role in generating the correct exceptional/atypical pronunciation of a written word. The TMS-induced decline in accuracy was specific in terms of both the stimulation site and the task (plus a marginally significant site-by-TMS interaction in surface errors). Another verbal production task (Repetition: Fig. 1) was not disrupted by rTMS on ATL, and rTMS to SMG did not lead to disruption in exception/atypical word reading. Also, the order effect of conditions/materials (i.e., a practice effect) did not explain the results (see Supplementary data).

Our rTMS-ATL targeted the focal area which various neuroimaging and neuropsychological studies suggest plays a crucial role in lexical-semantic processing. Cognitive psychology and computational modeling literatures have long debated what kind of item-specific information, semantic or lexical, has a causal role in the computation of atypical word pronunciation during reading. Past analyses of the performances in the semantic variant of PPA suggest that the role of semantic representations in ATL (Patterson et al. 2006; Woollams et al. 2007). Our Japanese-reading dual-pathway computational model also successfully simulated the rTMS effect on accuracy and error patterns by lesioning its semantic activations. Note that the model was not constructed in a way that semantic processing must support only exception/atypical word reading: Rather, the model was entirely free to use only the pathway via the phonetic input layer for all the word types. Also, the systematicity between the orthography and semantics in Japanese kanji words are incorporated into the representations of the model. This can allow the model to utilize such a systematicity. However, the systematicity exists for all the word types, and therefore all the word types potentially had an equal chance to utilize this systematicity between orthography and semantics when the model learns to read. In fact, however, we found that the contribution of the semantically mediated pathway emerged during training particularly for atypical/exception word reading as predicted by triangle models of reading. Thus, our simulation data are in agreement with an account that argues for the role of semantics in atypical/exception word reading (Plaut et al. 1996; Harm and Seidenberg 2004; Woollams et al. 2007; Dilkina et al. 2008).

Of course, this does not rule out a potential role of a lexicon in reading exception/atypical words, as per suggested by Coltheart et al. (2001), and it is possible that the mental lexicon might be represented in the ATL, where our rTMS targeted. Also, as Coltheart et al. (2001) argues, semantic involvement in reading may not be compulsory (see, Taylor et al. 2013, for details). Related to this, there are a small number of case reports of patients who had intact reading ability for atypical/exception words in the face of semantic impairment (i.e., dissociation) (Blazely et al. 2005). These cases are not inconsistent with our principal finding, of course, which is that ATL plays a necessary role in reading of these words. They do, however, pose a potential challenge to theories that explain ATL involvement in terms of a specifically semantic contribution. It is noteworthy that these occasional cases could be explained in terms of individual differences in the degree to which people rely on the semantic reading route. Indeed, computational models which incorporated such individual difference factors (e.g., writing/reading experience) have demonstrated that just such a dissociation is predicted in occasional individual cases, even though a strong association between semantic ability and exception/atypical words reading is present at the group level (Woollams et al. 2007; e.g., Dilkina et al. 2008).

Finally, our rTMS data do not rule out the role of other areas within temporal lobe in reading exception/atypical words, for example, the posterior part of the temporal lobe (Binder et al. 2016). Specifically, Binder et al. (2016)’s large-scale study with 45 focal stroke patients also found that lesions in the posterior temporal lobe were correlated with regularization reading errors. However, Binder et al. (2016) themselves acknowledge lesion coverage in their sample was limited to areas typically damaged in middle cerebral artery stroke, and did not include ventral temporal lobe or temporal pole, and thus they did not rule out the involvement of the ATL. We also would like to note that a recent study used direct electrical cortical stimulation and showed the stimulation in the visual word form area (i.e., posterior temporal lobe) slowed RT for single letter reading to the equivalent degree as RT for word reading (Hirshorn et al. 2016). Thus, it is more likely that this area has a broader role in reading, and less likely that it has a specific role for words with exception/atypical spelling-to-sound correspondences.

The role of the ATL in exception word reading can be interpreted in terms of dual dorsal–ventral frameworks in the brain, which are advocated in various cognitive domains (Poljak 1926; Ungerleider and Mishkin 1982; Hickok and Poeppel 2007). Typically, the ventral pathway is more relevant to computation of lexical/semantic information. Neuroanatomical models for reading already assume the existence of dual pathways (Pugh et al. 2000; Wilson et al. 2009; Graves et al. 2010; Hoffman et al. 2015; Binder et al. 2016), but the current study can refine this neuroanatomical theory. Specifically, some dorsal–ventral neuroanatomical models for reading postulate posterior temporal lobe involvement in whole-word reading, as part of a ventral pathway that passes through posterior inferior temporal lobe and the parietal lobe, but does not involve the ATL (Richlan 2012). Instead, we propose that the ventral pathway originates in posterior occipitotemporal cortex, passes through the ATL to terminate in the frontal lobe (Hoffman et al. 2015).

Having established the critical role of the ATL as part of the ventral reading pathway, it now becomes important to investigate how the ventral and dorsal pathways interact. Our model predicts that there is a division of labor between dorsal and ventral pathways, such that weakness in one is counterbalanced by strength in the other (for some evidence of this in functional neuroimaging, see (Seghier et al. 2008; Hoffman et al. 2015)). Combined rTMS/fMRI studies could play an important role in testing these predictions: disruption of the ventral pathway with ATL rTMS should result in an upregulation in activity in dorsal pathway regions. Studies of this kind have the potential to provide simultaneous data on the behavioral and neural consequences of TMS, and elucidate the network-level processes that give rise to impairments in exception word reading observed in neuropsychological populations. In sum, our rTMS result clearly supports the involvement of the ATL in exception word reading.

## Supplementary Material

Supplementary Data
