# Supplementary material for: The Ventral Anterior Temporal Lobe has a Necessary Role in Exception Word Reading
Source: Cereb Cortex. 2018 Jun 6;28(8):3035–45. doi: 10.1093/cercor/bhy131 (PMC6041960; doi:10.1093/cercor/bhy131)
Supplement: Supplementary Data [file bhy131_supplementarymaterials.zip › bhy131_Supplementary_Ueno_TMS_ATL_Reading.pdf]

Supplementary figures

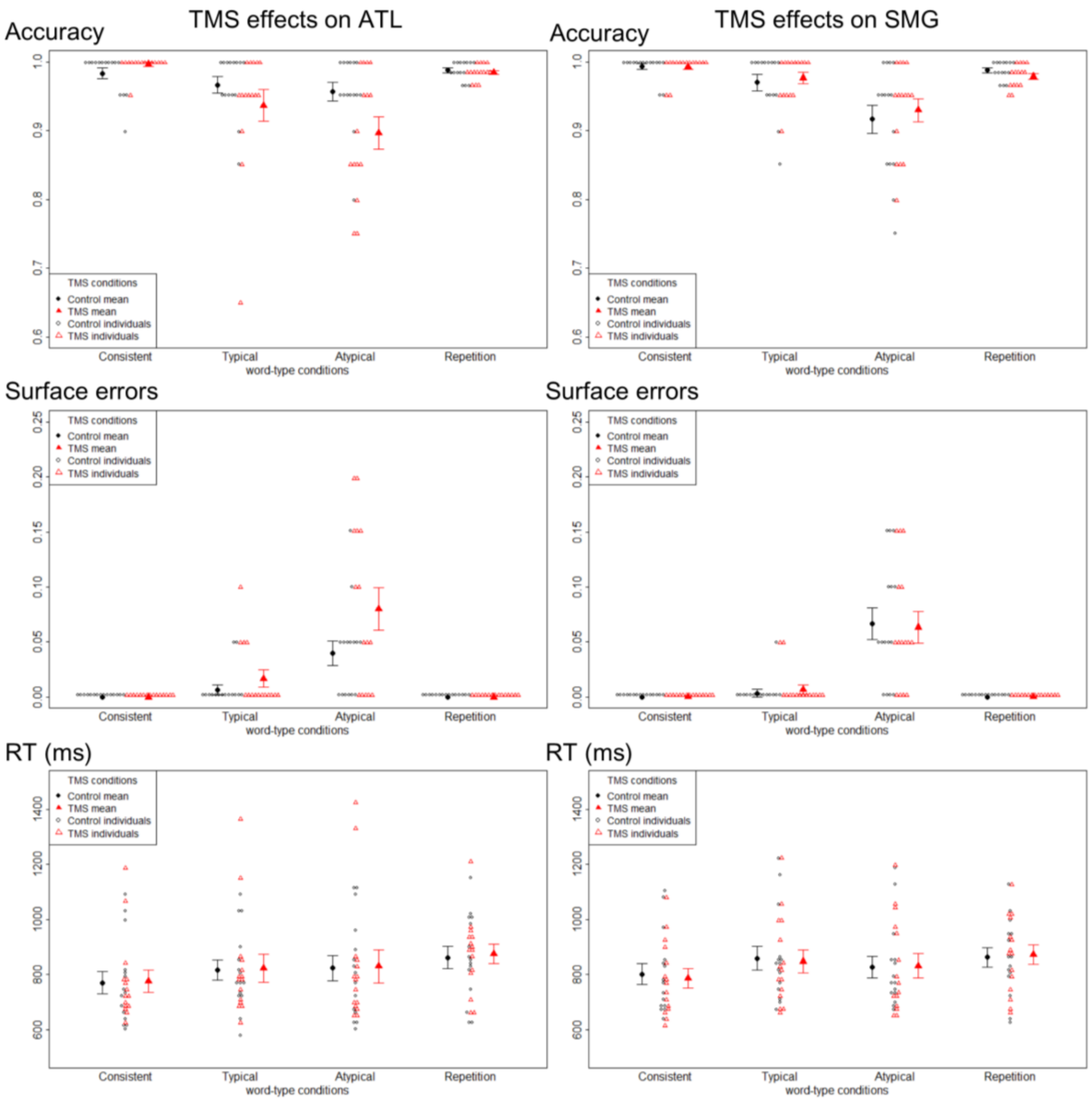

Figure S1. Individual data plots for accuracy (top row), surface errors (middle row), and reaction time (bottom row) as a function of the TMS sites (left column, ATL stimulation; right column, SMG stimulation) in the main experiment. Circles (black): control condition; triangles (red): TMS conditions.

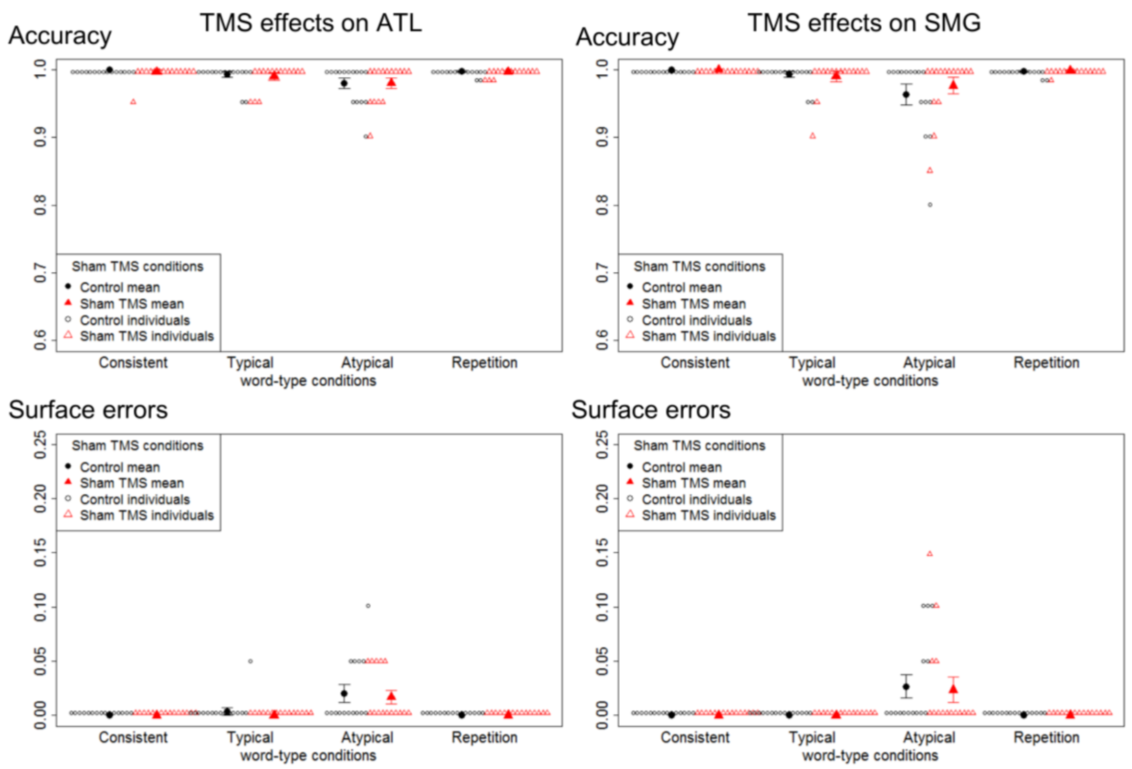

Figure S2. Individual data plots for accuracy (top row), and surface errors (bottom row), as a function of the "TMS" sites (left column, "ATL" stimulation; right column, "SMG" stimulation) in the control experiment. Circles (black): control condition; triangles (red): "TMS" conditions. A TMS pulse was not delivered in the "TMS" conditions. No effect of "TMS" was found in this control experiment.
